# Tracking control of air flow based on a fractional-order model of the lung impedance

**DOI:** 10.1038/s41598-024-77654-6

**Published:** 2025-10-07

**Authors:** Hadamez Kuzminskas, Marcelo Carvalho Minhoto Teixeira, Roberto Kawakami Harrop Galvão, Edvaldo Assunção, Sillas Hadjiloucas

**Affiliations:** 1 Department of Electrical Engineering, School of Engineering, São Paulo State University (UNESP), Ilha Solteira, SP 15385-007 Brazil; 2 Electronics Engineering Division, Instituto Tecnológico de Aeronáutica (ITA), São José dos Campos, SP 12228-900 Brazil; 3 Department of Biomedical Engineering, School of Biological Sciences, The University of Reading, Reading, RG6 6AY UK

**Keywords:** Biomedical engineering, Electrical and electronic engineering

## Abstract

A fractional order output feedback controller for a lung ventilator is designed. This is based on a state-of-the-art electrical analogue model of the human respiratory system in the form of a network of resistors and fractional capacitors. The electrical input impedance of the adopted analogue can be suitably tuned to fit experimental ventilation impedance data. Furthermore, it can explicitly account for the different physiological fractal type characteristics associated with lung formation such as branching morphogenesis associated to the treelike tubular network and alveolar differentiation associated with the generation of specialized epithelial cells for gas exchange. A description of this electrical analogue in pseudo-state space is then proposed. The aim is to finally provide a control methodology within the scope of output feedback control, when the measured output which is the airflow through the trachea is directed to follow a specified reference. The control provides adequate air pressure input to generate this nominal airflow. The proposed control design includes a pseudo-state observer and a double leaky integrator. The gains involved are designed using constraints imposed through linear matrix inequalities (LMIs), which enforce a regional allocation of eigenvalues. The robustness of the control loop is analysed through an uncertainty matrix analysis linked directly to the model. It is observed that the proposed design can tolerate a relatively wide variation in physiological parameters ($\pm 15\%$). The proposed formulation advances current control design approaches for mechanical ventilators and provides a generic methodology for the control of complex system with emergent responses as encountered in bioengineering.

## Introduction

Over the years, there have been several types of ventilators developed for short term intervention such as during anaesthesia in patients having normally functioning lungs. Typically, controllers were based on mean expired CO$_2$^1^, end-tidal CO$_2$^2^, arterial CO$_2$^3^ or pH^4^. It is usually the case that inspiratory pressure, tidal volume or mechanical rate or both are the control variables. However, it has been argued^5^ that end-tidal controllers do not take into account physiological deadspace ventilation as associated with patients with lung emboli. Furthermore, there are cases where hypoxemia may be observed during one-lung ventilation (OLV) as perfusion of the nonventilated lung affects oxygenation because air trapping in the ventilated lung may generate auto-positive end-expiratory pressure (PEEP)^6^. In OLV, air trapping in the nonventilated lung may delay the onset of desaturation. Currently there are two strategies to decrease the likelihood of hypoxemia, and possibly atelectasis during OLV, either using a high tidal volume (10–12 ml/kg) without PEEP^7^ or a moderate tidal volume (6–8 ml/kg) with PEEP^8^. In all the above cases, if a transient hypoperfusion of the lung is adopted, this would result in a decrease in end-tidal CO2 which is undesirable. Such aspects complicate controller design.

Focusing on recent advances in pressure support ventilation^9^, these have led to controller designs that use respiratory rate^10^, tidal volume^11^ or cycling pressure^12^ as control variables. Such controllers have a broader range of applications as they can be adapted to cater for totally paralyzed patients and spontaneous breathing subjects as well as support mixed modes of breathing. Although such strategies have their own merits, simple pressure support ventilation has been particularly promising for the provision of patient care in under-resourced regions or during pandemics such as COVID-19 with simple low-cost designs already under development or at the evaluation stage^13^. Furthermore, such solutions may be also used in proportional modes of ventilation^14^, where the aim is to amplify the effort of the patient’s respiratory muscle activity, providing the necessary support to improve the imbalance between capacity and demand and to reach the patient’s ventilation goal at the same time, as part of non-invasive ventilatory support, to control high-flow nasal oxygen as first-line treatment of acute hypoxemic respiratory failure and acute respiratory distress syndrome (ARDS)^15^.

An important issue in the design of controllers for ventilation systems is the development of accurate dynamic models for the lung mechanics. As discussed by Morton et al.^16^, there are two main types of lung models: complex descriptions based on finite element analysis and simpler lumped-parameter representations in the form of ordinary differential equations. Some models include aspects of lung morphology, surfactant properties, embedding of the alveoli in the lung tissue and mechanical characteristics of the chest wall^17^. Recently, an alternative approach based on the use of fractional-order models has emerged. Indeed, as highlighted by Magin^18^, there are several emerging areas (e.g., in studies of structure and function associated with neural anatomy, electrochemical biomolecular responses to excitatory signals, as well as in cell and tissue engineering studies of form and function) in which fractional-order models more accurately describe the dynamic response of living organisms to electrical, mechanical and chemical stimuli. Along this line, arguments regarding form and function have been considered based on the anatomy of the lung.

Historically, Weibel^19^ first used fractal geometry for the characterization of lung morphology, in order to study the aerodynamics of the organ. This and other past studies on lung geometry and morphology are currently being further validated using 3*D* computed tomography^20,21^. The healthy human respiratory system can be approximated by a quasi-symmetric structure, with 24 dichotomously bifurcated levels, in which the airflow is considered equally divided at each bifurcation. For each airway segment, an equivalent electrical impedance can be considered, in which the air flow and pressure are analogous to an electrical current and voltage, respectively. Impedance, which appears in analogy with electrical circuits, is capable of reproducing equivalent biomechanical responses^22–25^. Thus, a linear hydrodynamic model that describes variations in respiratory pressure and flow may be considered on the basis of an electrical transmission line analogue, whose electrical parameters of resistance, inertance and compliance can be obtained^22^. Such parameters may be further adjusted to account for variability for the individual branches of the tree^22^. Furthermore, it should be possible to account for different physiological characteristics associated with lung formation such as branching morphogenesis associated to the treelike tubular network as well as alveolar differentiation associated with the generation of specialized epithelial cells for gas exchange.

In this same line of research, subsequent work demonstrated the link between the recurrence of the respiratory tree and the appearance of fractional order: these were further evidenced by the appearance of a constant phase response in the total respiratory impedance as a consequence of the intrinsic fractal geometry of the lungs^23^. Furthermore, a previous study concluded that the fractional-order model outperforms the integer-order one in terms of characterizing the dependence of the input impedance of the human respiratory model on frequency^26^. Its main advantages over integer-order models are the smaller number of parameters and its inherent ability to characterize the viscoelastic properties and recurrent structures of biological materials^27,28^. A fractional-order model can be implemented with integer-order approximations over a specified frequency range, but this results in higher-order transfer functions^29^. The subject has been in a constant state of evolution with the introduction of the modelling of viscous losses in the representation of the electrical equivalent, which become relevant with the pathological increase of the heterogeneity of the pulmonary parenchyma^24^. It is worth mentioning that such approaches comprised only elastic components and were not able to capture changes in respiratory impedance with the occurrence of diseases.

The basis for the present work is the approach of Ionescu et al.^25^, in which the fractional order is modelled directly in a ladder network representation of the respiratory tree through the use of a Constant Phase Element (CPE). This element, also known as fractional order capacitor, has impedance $Z_{CPE}={1}/({s^{\alpha }C})$, where *C* is the pseudo-capacitance^30^ and $\alpha >0$ is the fractional order. Such a modelling approach has physiological origin and is verifiable experimentally using a forced oscillation technique. At this point, it is worth mentioning that it is also possible to assume a lumped model that is capable of producing the same tissue impedance but over a more restricted frequency domain. Such model, however, is likely to have much more limited applications in healthcare^31^. Indeed, in the study conducted by Ionescu et al.^25^ with data from seven subjects, the ladder-type models provided a better fit compared to the use of lumped models. It is also worth noting that recursive network models can allow significant variations of model parameters in relation to frequency, and thus can be more useful in emulating the impedance over a wide range of frequencies^32^.

**Contribution:** The development of control laws for mechanical ventilators has been an active research area. For example, D’Orsi et al.^33^ employed a predictive control strategy to manipulate the applied pressure in order to maximize arterial oxygen saturation while avoiding barotrauma. Almeida et al.^34^ developed a strategy based on Active Disturbance Compensation Control with volume and pressure control modes. The proposed technique incorporated robust state observers to handle external disturbances and parametric uncertainties. Violini et al.^35^ employed a gain-scheduling scheme with pressure and flow compensation to achieve a desired PEEP level in the expiration phase. Reinders et al.^36^ proposed the use of a repetitive control technique employing a Lur’e-type model to take into account the nonlinear resistance of the ventilation hose. In this context, the novelty of the present work consists in designing an output feedback controller based on a fractional-order model of the lung impedance^25^. The design method proposed herein is aimed at flow control^37^, p. 18, which can be used in an inner loop to achieve accurate control of volume and pressure^38^. The control design includes a pseudo-state observer and a double leaky integrator^39^. The gains involved are designed through conditions in linear matrix inequality (LMI) form for the regional allocation of eigenvalues. The robustness of the control loop is analysed through uncertain matrices attached directly to the model, which account for variations in parameters within percentage ranges.

**Text organization:** Section “Fractional-order model of the lung impedance” presents the electrical analogue model for the human respiratory system and derives an alternative mathematical representation in pseudo-state space. Section “[Sec Sec3]” proposes LMI conditions for the regional allocation of eigenvalues in a specified region. Section “Output feedback control employing a pseudo-state observer” proposes an output control method that uses a pseudo-state observer and a double leaky integrator, with gains obtained from the regional allocation of eigenvalues. Section “Results” presents the results of the proposed control methodology applied to the electrical analogue of the model for the human respiratory system. Section “Conclusions” presents the concluding remarks. **Mathematical notations:** $\vert z\vert$ is the absolute value of a real number *z*; *arg*(*z*) is the argument of the complex number *z*; $M^{T}$ is the transpose of *M*; $M_{n\times n}$ is the matrix *M* of dimension $n\times n$; $I_{n\times n}$ is the identity matrix of dimension $n\times n$; $0_{n\times n}$ is the zero matrix of dimension $n\times n$; $\overline{M}$ is the complex conjugate of *M*; $A\succ B$ if and only if $A-B$ is a positive-definite matrix; $A\prec B$ if and only if $A-B$ is a negative-definite matrix; $\lambda (M)$ is the set of eigenvalues of the matrix *M*; *F*, *S* denote regions of interest in the complex plane.

## Fractional-order model of the lung impedance

**Fig. 1:**
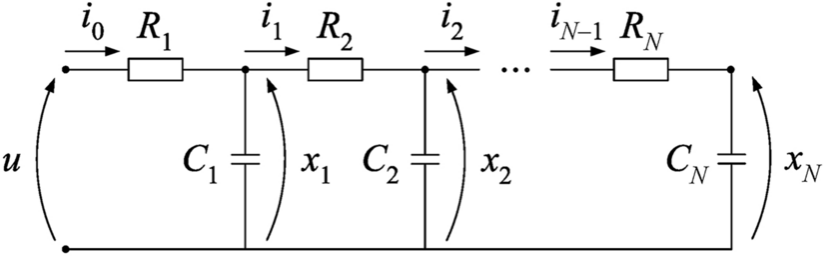
Network model.

Figure 1 presents the ladder network, corresponding to the electrical analogue of the human respiratory system, employed by Ionescu et al.^25^, which has $N = 24$ stages. The capacitor symbols indicates a constant phase element (CPE)^30^ with impedance $Z_m(s) = 1/(C_m s^\alpha )$, $m = 1, 2,\ldots ,N$.

By using the pseudo-state variables indicated in Fig. 1, a pseudo-state space model can be obtained on the basis of the following current-voltage relationships, where $\mathscr {D}^\alpha$ denotes the fractional derivative of order $\alpha \in (0,2)$:

1 $$\begin{aligned}&\begin{array}{ccccc} \mathscr {D}^\alpha x_1 = {\left( i_0 - i_1\right) }/{C_1},&\mathscr {D}^\alpha x_2 = {\left( i_1 - i_2\right) }/{C_2},&\cdots ,&\mathscr {D}^\alpha x_{N-1} = {\left( i_{N-2} - i_{N-1}\right) }/{C_{N-1}},&\mathscr {D}^\alpha x_{N} = {i_{N-1}}/{C_{N}}, \end{array} \end{aligned}$$

2 $$\begin{aligned}&\begin{matrix} i_0 = {\left( u - x_1\right) }/{R_1},&i_1= {\left( x_1 - x_2\right) }/{R_2},&\cdots ,&i_{N-2} = {\left( x_{N-2} - x_{N-1}\right) }/{R_{N-1}},&i_{N-1} = {\left( x_{N-1} - x_{N}\right) }/{R_{N}}. \end{matrix} \end{aligned}$$

As in Ionescu et al.^25^, the resistance and capacitance values are given by $R_m = {R^*_m}/{2^{m-1}}$, $C_m = 2^{m-1} C_m^*$, $m = 1, 2, \ldots , 24$, where $R^*_m$ and $C^*_m$ follow these recursive expressions, with $R_{UA}$ and $C_{UA}$ corresponding to the upper airways up to the trachea:

3 $$\begin{aligned} \begin{matrix} \left\{ \begin{matrix} R_1^*= R_{UA}, \\ C_1^*= C_{UA}, \end{matrix} \right. & \begin{matrix} \left\{ \begin{matrix} R_{m+1}^*= 2.476 R_m^*, \\ C_{m+1}^*= 0.577 C_m^*, \end{matrix} \right. & m = 1, 2, \ldots , 13, \end{matrix} & \begin{matrix} \left\{ \begin{matrix} R_{m+1}^*= 2.334 R_m^*, \\ C_{m+1}^*= 0.613 C_m^*, \end{matrix} \right.&m = 14, 15, \ldots , 23. \end{matrix} \end{matrix} \end{aligned}$$

Herein, we shall employ the first set of $R_{UA}$, $C_{UA}$, and $\alpha$ values experimentally obtained by Ionescu et al.^25^, which were identified on the basis of forced oscillation data^40^ in the range from 0.9 to 6 Hz. The reported parameters were

4 $$\begin{aligned} R_{UA} = 0.2834~\text {[kPa s/l]},\,&C_{UA} = 0.0950~\text {[l s}^{\alpha -1}\text {/kPa]},&\alpha = 1.55. \end{aligned}$$

Relations (1) and (2) lead to

5 $$\begin{aligned}& \underbrace{ \begin{bmatrix} \mathscr {D}^\alpha x_1 \\ \mathscr {D}^\alpha x_2 \\ \mathscr {D}^\alpha x_3 \\ \vdots \\ \mathscr {D}^\alpha x_{N-1} \\ \mathscr {D}^\alpha x_N \\ \end{bmatrix}}_{\mathscr {D}^\alpha x} \\&= \underbrace{ \begin{bmatrix} -\dfrac{1}{R_1C_1} -\dfrac{1}{R_2C_1} & \dfrac{1}{R_2C_1} & 0 & \cdots & 0 & 0 \\ \dfrac{1}{R_2C_2} & -\dfrac{1}{R_2C_2} -\dfrac{1}{R_3C_2} & \dfrac{1}{R_3C_2} & \cdots & 0 & 0 \\ 0 & \dfrac{1}{R_3C_3} & -\dfrac{1}{R_3C_3} -\dfrac{1}{R_4C_3} & \cdots & 0 & 0 \\ \vdots & \vdots & \vdots & \ddots & \vdots & \vdots \\ 0 & 0 & 0 & \cdots & -\dfrac{1}{R_{N-1}C_{N-1}} -\dfrac{1}{R_{N}C_{N-1}} & \dfrac{1}{R_N C_{N-1}}\\ 0 & 0 & 0 & \cdots & \dfrac{1}{R_N C_N} & -\dfrac{1}{R_N C_N} \\ \end{bmatrix} }_A \underbrace{ \begin{bmatrix} x_1 \\ x_2 \\ x_3 \\ \vdots \\ x_{N-1} \\ x_N \\ \end{bmatrix} }_x + \underbrace{ \begin{bmatrix} \dfrac{1}{R_1C_1} \\ 0 \\ 0 \\ \vdots \\ 0 \\ 0 \\ \end{bmatrix} }_B u. \end{aligned}$$

The output equation for the measured variable is expressed as

6 $$\begin{aligned} \begin{matrix} y = i_{0}={(u - x_1)}/{R_1} = Cx + Du,&C=\begin{bmatrix} -{1}/{R_1}&0&0&\cdots&0&0\end{bmatrix},&D=\begin{bmatrix}{1}/{R_1}\end{bmatrix}. \end{matrix} \end{aligned}$$

### Remark 1

The vector *x*(*t*) in (5) does not strictly represent the state vector, it is referred to in the literature as the pseudo-state vector. The true state vector has infinite dimension and its components are internal to the integrators^41–43^.

## $\mathfrak {D}$-stability condition for fractional order systems

LMIs are widely used in system analysis and synthesis of efficient controllers. The convexity property is one of its advantages, which enables the use of well-established numerical optimization methods. Another advantage is the ability to simultaneously contemplate several constraints related, for example, to physical limits of the system, stability, performance and robustness^44^. One of the desirable restrictions to the closed-loop system is the $\mathfrak {D}$-stability, which consists in the allocation of eigenvalues in a specific region of the complex plane, in order to guarantee certain performance indices of the responses involved. Conditions in LMIs for $\mathfrak {D}$-stability go back to Chilali et al.^45,46^, whose fundamental ideas were later applied to fractional systems^47,48^. In addition to the mentioned results, there is a variety of works that provide conditions in LMIs in the general scope of stability and control of fractional systems^49–52^.

A fundamental result concerning the stability of fractional-order systems is stated in Theorem 1 below.

### Theorem 1

^49,50^ *Consider a linear fractional-order system of the form*

7 $$\begin{aligned} \mathscr {D}^\alpha x(t) = Ax(t) \,,\; \alpha \in (0,2), \end{aligned}$$

*where* $\alpha$* is the differentiation order,* $x(t)\in \mathbb {R}^n$* is the pseudo-state vector and* $A\in \mathbb {R}^{n\times n}$ *is a constant matrix. System* (7) * is asymptotically stable if and only if all eigenvalues of A are located in the region* $F(\alpha )$ *defined as* $F(\alpha ) = \left\{ \lambda \in \mathbb {C}: |\text {arg}(\lambda )| > \frac{\alpha \pi }{2} \right\}$.

**Fig. 2:**
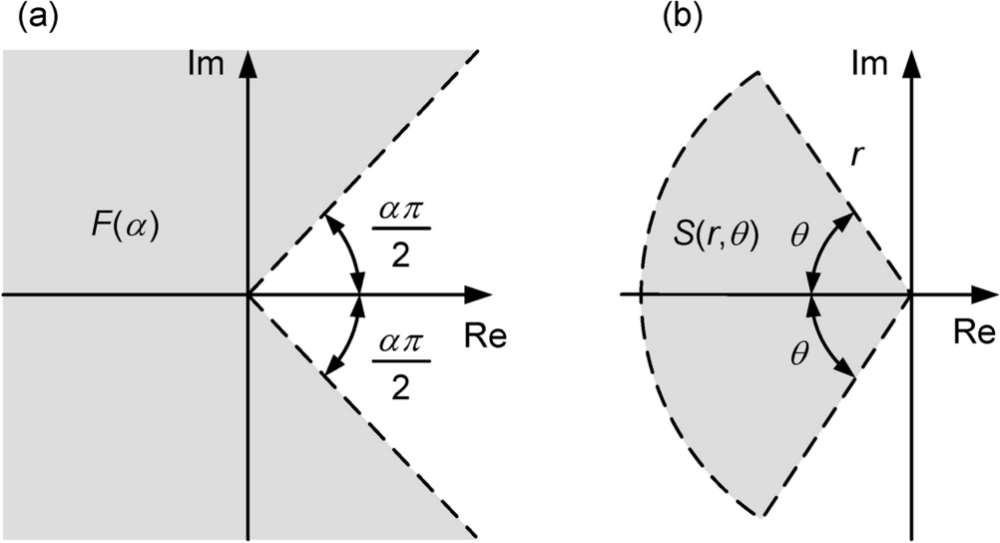
(**a**) Stability region $F(\alpha )$ and (**b**) region $S\left( r,\theta \right)$ for eigenvalue allocation.

The stability region $F(\alpha )$ is depicted in Fig. 2a. In what follows, the concept of $\mathfrak {D}$-stability is exploited to characterize a convenient subset of this region in terms of LMI conditions.

### Definition 1

^45,46^ A region $\mathfrak {D}$ of the complex plane is called an LMI region if there exists a symmetric matrix $L\in \mathbb {R}^{m\times m}$ and a matrix $M\in \mathbb {R}^{m\times m}$ such that $\mathfrak {D}=\left\{ z\in \mathbb {C}: f_{\mathfrak {D}}(z)\prec 0\right\}$, where $f_{\mathfrak {D}}(z)=L+zM+\bar{z}M^{T}$ is called the characteristic function of $\mathfrak {D}$.

### Definition 2

^45,46^ A matrix $A\in \mathbb {R}^{n\times n}$ is said to be $\mathfrak {D}$-stable if all its eigenvalues are contained in $\mathfrak {D}$.

Herein, we shall be concerned with an LMI region defined as $S\left( r,\theta \right) = \{ z \in \mathbb {C} : \; f_r(z)\prec 0,f_\theta (z)\prec 0\}$ with

8 $$\begin{aligned}&f_r(z)=\begin{bmatrix}-r& 0\\ 0& -r\end{bmatrix}+z\begin{bmatrix}0& 1\\ 0& 0\end{bmatrix}+\bar{z}{\begin{bmatrix}0& 1\\ 0& 0\end{bmatrix}}^{T}=\begin{bmatrix}-r& z\\ \bar{z}& -r\end{bmatrix}, \end{aligned}$$

9 $$\begin{aligned}&f_\theta (z)=z\begin{bmatrix}sin(\theta )& cos(\theta )\\ -cos(\theta )& sin(\theta )\end{bmatrix}+\bar{z}\begin{bmatrix}sin(\theta )& cos(\theta )\\ -cos(\theta )& sin(\theta ) \end{bmatrix}^{T} = \begin{bmatrix}\left( z+\bar{z}\right) sin(\theta )& \left( z-\bar{z}\right) cos(\theta )\\ -\left( z-\bar{z}\right) cos(\theta )& \left( z+\bar{z}\right) sin(\theta ) \end{bmatrix} \end{aligned}$$

where $r > 0$ and $0 < \theta \le \pi /2$. The inequalities $f_r(z)\prec 0$, and $f_\theta (z)\prec 0$ correspond to the disk centered at the origin with radius *r* and to the conical sector in the left half-plane with vertex at the origin and interior angle $2\theta,$ respectively  ^45,46^, as shown in Fig. 2b.

### Remark 2

If the eigenvalues of *A* lie in $S(r, \theta )$ and $S(r, \theta ) \subset F(\alpha )$, the fractional order system (7) will be asymptotically stable. If $\alpha \in (0,1]$, the condition $S(r, \theta ) \subset F(\alpha )$ will hold for any $\theta \in (0, \pi /2]$. If $\alpha \in (1,2)$, $\theta$ will have to be chosen in the range $(0, \pi - \alpha \pi /2]$.

Necessary and sufficient LMI conditions for the eigenvalues of *A* to lie in $S(r, \theta )$ are given in Lemma 1 below.

### Lemma 1

*The eigenvalues of a matrix* $A \in \mathbb {R}^{n \times n}$ *will lie in* $S(r, \theta )$ *if and only if there is a positive-definite matrix* $X = X^T \in \mathbb {R}^{n\times n}$ *such that*

10 $$\begin{aligned}&\begin{bmatrix} -rX& AX \\ XA^{T}& -rX \end{bmatrix} \prec 0, \end{aligned}$$

11 $$\begin{aligned}&\begin{bmatrix} \left( AX+XA^{T}\right) sin(\theta )& \left( AX-XA^{T}\right) cos(\theta ) \\ \left( -AX+XA^{T}\right) cos(\theta )& \left( AX+XA^{T}\right) sin(\theta ) \end{bmatrix}\prec 0. \end{aligned}$$

### Proof

Follows directly from the necessity and sufficiency of the LMIs numbered as (10) and (13) in the paper by Chilali and Gahinet^45^. $\square$

Now, consider a linear fractional order system of the form (7) augmented with a control input $u(t)\in \mathbb {R}^{p}$, i.e. $\mathscr {D}^{\alpha }x(t) = Ax(t) + Bu(t)$, where $B\in \mathbb {R}^{n\times p}$. By using a feedback control law $u(t)=-Kx(t)$ with $K \in \mathbb {R}^{p \times n}$, the closed-loop dynamics become

12 $$\begin{aligned} \mathscr {D}^{\alpha }x(t)=\left( A-BK\right) x(t). \end{aligned}$$

As stated in Theorem 2 below, a gain matrix *K* that places the eigenvalues of $(A-BK)$ in the region $S(r, \theta )$ can be obtained from the solution of an LMI feasibility problem.

### Theorem 2

*If there is a positive-definite matrix* $X=X^{T}\in \mathbb {R}^{n\times n}$ *and a matrix* $M\in \mathbb {R}^{p\times n}$ *such that*

13 $$\begin{aligned}&\begin{bmatrix} -rX& AX-BM \\ XA^{T}-M^{T}B^{T}& -rX \end{bmatrix} \prec 0, \end{aligned}$$

14 $$\begin{aligned}&\begin{bmatrix} \left( AX+XA^{T}-BM-M^{T}B^{T}\right) sin(\theta )& \left( AX-XA^{T}-BM+M^{T}B^{T}\right) cos(\theta )\\ \left( -AX+XA^{T}+BM-M^{T}B^{T}\right) cos(\theta )& \left( AX+XA^{T}-BM-M^{T}B^{T}\right) sin(\theta ) \end{bmatrix}\prec 0, \end{aligned}$$

*then the eigenvalues of* $(A-BK)$ *with* $K = MX^{-1}$ *will lie in* $S(r, \theta )$ .

### Proof

Assume that (13) and (14) hold. Replacing *KX* for *M*, it follows that

15 $$\begin{aligned}&\left[ \begin{matrix} -rX& \left( A-BK\right) X \\ X\left( A-BK\right) ^{T}& -rX \end{matrix} \right] \prec 0, \end{aligned}$$

16 $$\begin{aligned}&\left[ \begin{matrix} (\left( A-BK\right) X+X\left( A-BK\right) ^{T})sin(\theta )& (\left( A-BK\right) X-X\left( A-BK\right) ^{T})cos(\theta )\\ (-\left( A-BK\right) X+X\left( A-BK\right) ^{T})cos(\theta )& (\left( A-BK\right) X+X\left( A-BK\right) ^{T})sin(\theta ) \end{matrix} \right] \prec 0, \end{aligned}$$

which correspond to (10), (11) with $(A-BK)$ in place of *A*. Therefore, it follows from Lemma 1 that the eigenvalues of $(A-BK)$ will lie in $S(r, \theta )$. $\square$

### Remark 3

If the pair (*A*, *B*) is controllable^53,54^, there will always be a feasible solution $X \succ 0$, *M* to the LMIs (13), (14). Indeed, under the controllability assumption, it is always possible to find a matrix *K* such that the eigenvalues of $(A-BK)$ lie in $S(r, \theta )$. The feasibility property follows by replacing *M* with *KX* and noting that Lemma 1, with $(A-BK)$ in place of *A*, ensures the existence of $X \succ 0$ such that (13), (14) hold.

## Output feedback control employing a pseudo-state observer

**Fig. 3:**
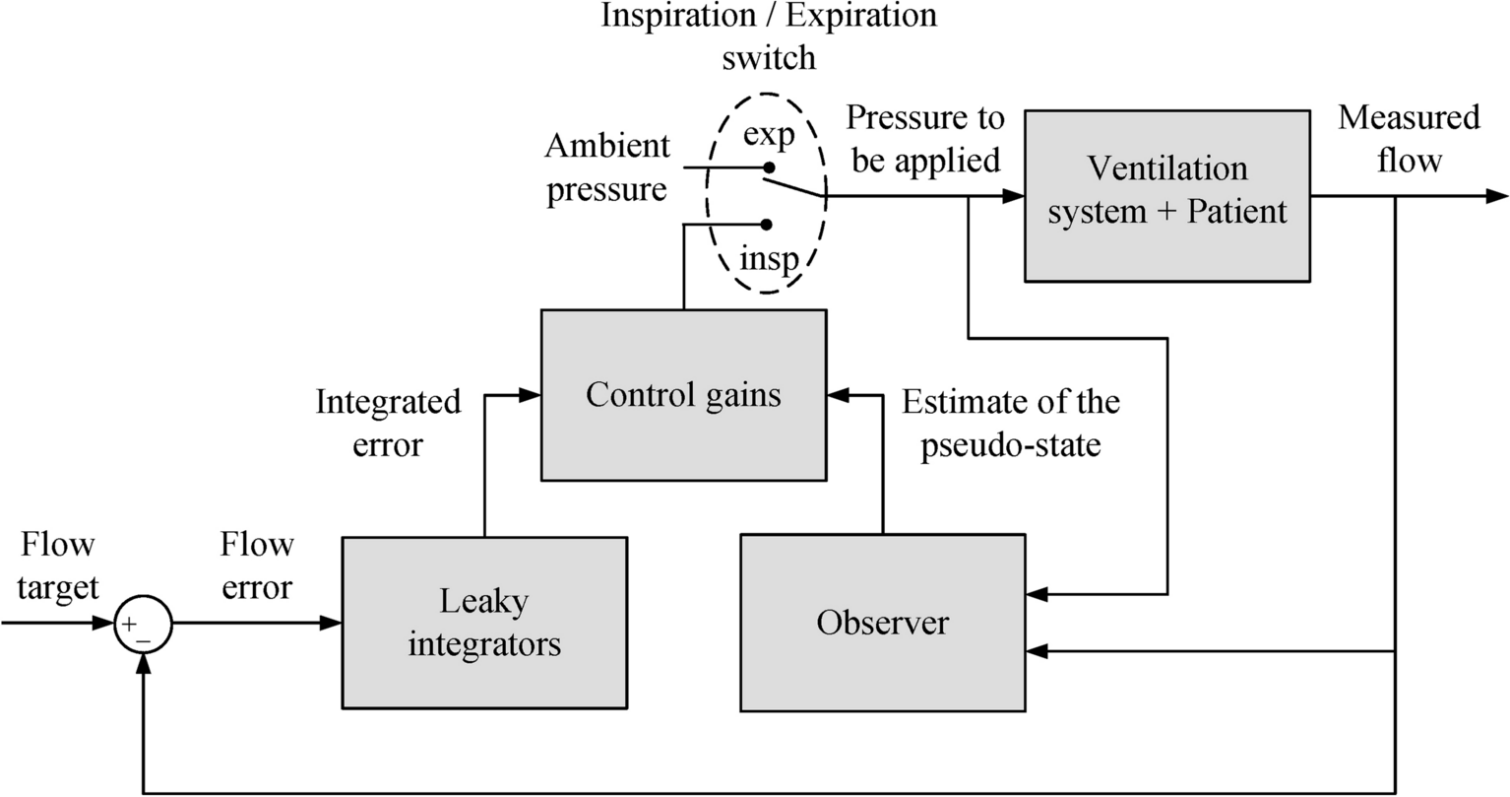
Main components of the proposed controller.

**Fig. 4:**
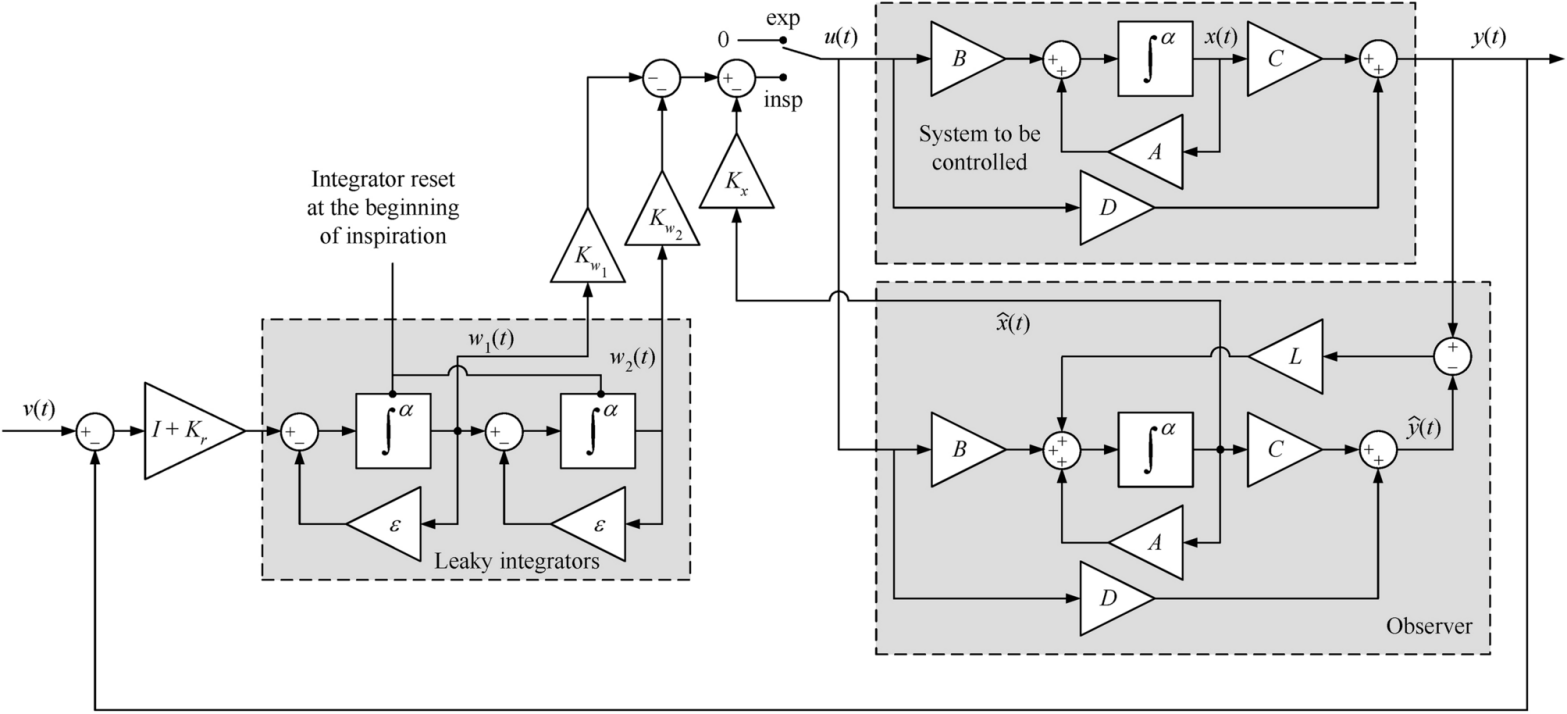
Detailed description of the closed-loop system in block diagram form.

The general approach adopted is based on further extension of formulations encountered in classical control theory^55^ to fractional order systems. In practical applications, the pseudo-states may not be fully accessible, only the inputs and outputs of the fractional plant being known. In this case, the estimation of the pseudo-states and the control of the plant can be obtained through a pseudo-state observer. Several works based on the Luenberger-type fractional-order linear observer are reported in the literature, with different scopes such as robust stabilization, for example^56–58^. It is assumed that the system under consideration is described by a model of the form

17 $$\begin{aligned} \mathscr {D}^\alpha x(t)&= A x(t) + B u(t), \end{aligned}$$

18 $$\begin{aligned} y(t)&= Cx(t) + Du(t), \end{aligned}$$

where $\alpha \in (0,2)$ is the differentiation order, $x(t)\in \mathbb {R}^n$ is the pseudo-state vector, $u(t)\in \mathbb {R}^p$ is the input, $y(t)\in \mathbb {R}^q$ is the output vector and $A\in \mathbb {R}^{n\times n}$, $B\in \mathbb {R}^{n\times p}$, $C\in \mathbb {R}^{q\times n}$ and $D\in \mathbb {R}^{q\times p}$ are constant matrices. Moreover, it is assumed that the output *y*(*t*) is measured. An output feedback controller with double integral action is proposed below, in which the pseudo-state estimation is performed through the use of a Luenberger-type fractional-order linear observer:

19 $$\begin{aligned} \mathscr {D}^\alpha \hat{x}(t)&= A \hat{x}(t) + B u(t) + L(y(t) - \hat{y}(t)), \end{aligned}$$

20 $$\begin{aligned} \hat{y}(t)&= C\hat{x}(t) + Du(t), \end{aligned}$$

21 $$\begin{aligned} \mathscr {D}^{\alpha } w_1(t)&= (I_{q \times q} + K_r) ( v(t) - y(t)) - \varepsilon \, w_1(t), \end{aligned}$$

22 $$\begin{aligned} \mathscr {D}^{\alpha } w_2(t)&= w_1(t) - \varepsilon \, w_2(t), \end{aligned}$$

23 $$\begin{aligned} u(t)&= -K_x \hat{x}(t) - K_{w_1} w_1(t) - K_{w_2} w_2(t), \end{aligned}$$

where $\hat{x}(t)\in \mathbb {R}^{n}$ is the observer pseudo-state vector, $\hat{y}(t)\in \mathbb {R}^q$ is the observer output, $w_{1}(t),w_{2}(t)\in \mathbb {R}^{q}$ are the integrator outputs, $v(t) \in \mathbb {R}^q$ is the reference signal to be tracked, $\varepsilon$ is a small positive constant and $K_r \in \mathbb {R}^{q \times q},$ $L\in \mathbb {R}^{n \times q}$, $K_x \in \mathbb {R}^{p \times n}$, $K_{w_1} \in \mathbb {R}^{p \times q}$, $K_{w_2}\in \mathbb {R}^{p \times q}$ are gain matrices to be adjusted. Figure 3 presents a block diagram of the main components of the control system, with a more detailed view depicted in Fig. 4. It is worth noting that the expiration phase is performed in a passive manner, i.e. the switch at the ventilator input is set to the ambient pressure during expiration. The only non-standard components in the proposed controller are the fractional-order integrators $\int ^{\alpha }$ in Fig. 4. However, these integrators can be easily realized by using analogue circuits^59^ or field-programmable gate arrays^60^.

### Remark 4

If a constant input *u* is applied to the ladder model in Fig. 1, the output $y = i_0$ will converge to a null steady-state value, which points to the presence of a zero at $s = 0$ in the network admittance. Therefore, double integral action is required to track a constant setpoint for the airflow without steady-state error^53,54^. However, if the control law employed pure integrators, a pole-zero cancelation would occur at $s = 0$ and the closed-loop system would be unstable, with an unbounded growth of the control signal when tracking the constant airflow setpoint. By using a small positive value for $\varepsilon$ in (21), (22) to introduce leakage terms in the fractional-order integrators^39^, the instability issue is avoided at the cost of allowing for some steady-state error in the tracking task. The value of $\varepsilon$ can be tuned to achieve a suitable trade-off between the magnitudes of the error and control signals. To this end, different scalars $\varepsilon _1$ and $\varepsilon _2$ could be employed in (21) and (22), respectively. Herein, we opted to use a single scalar $\varepsilon$ to facilitate the tuning procedure.

Let the pseudo-state estimator be

24 $$\begin{aligned} \tilde{x}(t) = x(t) -\hat{x}(t). \end{aligned}$$

From (24) and (23), the control law can be expressed as

25 $$\begin{aligned} u(t) = - K_x ( x(t) - \tilde{x}(t)) - K_{w_1} w_1(t) - K_{w_2} w_2(t). \end{aligned}$$

After replacing (25) for *u*(*t*) in (17), one arrives at

26 $$\begin{aligned} \mathscr {D}^\alpha x(t) = (A - BK_x) x(t) + BK_x \tilde{x}(t) - BK_{w_1} w_1(t) - BK_{w_2} w_2(t). \end{aligned}$$

Applying the $\mathscr {D}^\alpha$ operator on (24), from (17), (18), (19) and (20), leads to

27 $$\begin{aligned} \mathscr {D}^\alpha \tilde{x}(t) = \mathscr {D}^\alpha x(t) - \mathscr {D}^\alpha \hat{x}(t) = A (x(t) - \hat{x}(t)) - LC( x(t) - \hat{x}(t)) = (A-LC)\tilde{x}(t). \end{aligned}$$

Substituting (18) and (25) into (21) results in

28 $$\begin{aligned} \mathscr {D}^{\alpha } w_1(t)&= (I_{q \times q} + K_r)(v(t) - Cx(t)) + (I_{q \times q} + K_r)DK_x (x(t) - \tilde{x}(t)) \nonumber \\&\quad + (I_{q \times q} + K_r)DK_{w_1} w_1(t) + (I_{q \times q} + K_r)DK_{w_2} w_2(t) - \varepsilon w_1(t), \nonumber \\&= (I_{q \times q} + K_r) v(t) + (I_{q \times q} + K_r) (DK_x - C) x(t) - (I_{q \times q} + K_r)DK_x \tilde{x}(t) \nonumber \\&\quad + ((I_{q \times q} + K_r)DK_{w_1} - \varepsilon I_{q \times q}) w_1(t) + (I_{q \times q} + K_r) DK_{w_2} w_2(t). \end{aligned}$$

In light of (22), (26), (27) and (28), the closed-loop dynamics can be described by the following pseudo-state equation:

29 $$\begin{aligned} \left[ {\begin{array}{*{20}c} {{\mathcal{D}}^{\alpha } x(t)} \\ {{\mathcal{D}}^{\alpha } w_{1} (t)} \\ {{\mathcal{D}}^{\alpha } w_{2} (t)} \\ {{\mathcal{D}}^{\alpha } \tilde{x}(t)} \\ \end{array} } \right] = & \left[ {\begin{array}{*{20}c} {(A - BK_{x} )} & { - BK_{{w_{1} }} } & { - BK_{{w_{2} }} } & {BK_{x} } \\ {(I_{{q \times q}} + K_{r} )(DK_{x} - C)} & {(I_{{q \times q}} + K_{r} )DK_{{w_{1} }} - \varepsilon I_{{q \times q}} } & {(I_{{q \times q}} + K_{r} )DK_{{w_{2} }} } & { - (I_{{q \times q}} + K_{r} )DK_{x} } \\ {0_{{q \times n}} } & {I_{{q \times q}} } & { - \varepsilon I_{{q \times q}} } & {0_{{q \times n}} } \\ {0_{{n \times n}} } & {0_{{n \times q}} } & {0_{{n \times q}} } & {(A - LC)} \\ \end{array} } \right]\left[ {\begin{array}{*{20}c} {x(t)} \\ {w_{1} (t)} \\ {w_{2} (t)} \\ {\tilde{x}(t)} \\ \end{array} } \right] \\ & + \left[ {\begin{array}{*{20}c} {0_{{n \times q}} } \\ {I_{{q \times q}} + K_{r} } \\ {0_{{q \times q}} } \\ {0_{{n \times q}} } \\ \end{array} } \right]v(t). \\ \end{aligned}$$

Moreover, from (18) and (25), the output equation can be written as

30 $$\begin{aligned} y(t) = Cx(t) - D K_x (x(t) - \tilde{x}(t)) - D K_{w_1} w_1(t) - D K_{w_2} w_2(t) = (C - D K_x) x(t) + D K_x \tilde{x}(t) - D K_{w_1} w_1(t) - D K_{w_2} w_2(t). \end{aligned}$$

In view of (29), the closed-loop eigenvalues comprise the eigenvalues of $(A-LC)$ and $(A_{{xw}_r} - B_{{xw}_r}K_{xw})$, where

31 $$\begin{aligned} A_{{xw}_r} = \begin{bmatrix} A & 0_{n \times q} & 0_{n \times q} \\ -(I_{q \times q}+K_r) C & -\varepsilon I_{q \times q} & 0_{q \times q} \\ 0_{q \times n} & I_{q \times q} & -\varepsilon I_{q \times q} \end{bmatrix},&\,B_{{xw}_r} = \begin{bmatrix} B \\ -(I_{q \times q}+K_r)D \\ 0_{q \times p} \end{bmatrix},&K_{xw} = \begin{bmatrix} K_x & K_{w_1} & K_{w_2} \\ \end{bmatrix}. \end{aligned}$$

Given a gain matrix $K_r$, a stable closed-loop system can be obtained by choosing $K_{xw}$ and *L* such that the eigenvalues of $(A_{xwr} - B_{xwr}K_{xw})$ and $(A-LC)$ are located in region $F(\alpha )$ of Theorem 1. To this end, Theorem 2 can be employed to perform a regional allocation of the eigenvalues.

### Robustness analysis

The design procedure presented above does not provide formal guarantees of robust stability. The results could be extended to account for the use of uncertain design models, as described in Sections III-A and IV of the paper by Chilali and Gahinet^45^. However, in the present work, the robustness of the design will be evaluated by means of numerical simulations. Parametric changes are modelled using new matrices $\bar{A}$, $\bar{B}$, $\bar{C}$ and $\bar{D}$ having, respectively, the same dimensions of *A*, *B*, *C* and *D*:

32 $$\begin{aligned} \mathscr {D}^\alpha x(t)&= \bar{A} x(t) + \bar{B} u(t), \end{aligned}$$

33 $$\begin{aligned} y(t)&= \bar{C} x(t) + \bar{D}u(t). \end{aligned}$$

After replacing (25) for *u*(*t*) in (32), one arrives at

34 $$\begin{aligned} \mathscr {D}^\alpha x(t) = (\bar{A} - \bar{B}K_x) x(t) + \bar{B}K_x \tilde{x}(t) - \bar{B}K_{w_1} w_1(t) - \bar{B}K_{w_2} w_2(t). \end{aligned}$$

Moreover, after replacing (20) for $\hat{y}(t)$ and (33) for *y*(*t*) in (19), one arrives at

35 $$\begin{aligned} \mathscr {D}^\alpha \hat{x}(t) = A \hat{x}(t) + B u(t) + L(\bar{C} x(t) + \bar{D}u(t) - {C} \hat{x}(t) - {D}u(t)) = (A - LC) \hat{x}(t) + L \bar{C} x(t) + \big [B + L (\bar{D} - D)\big ]u(t). \end{aligned}$$

Again, consider the pseudo-state estimation error given by

36 $$\begin{aligned} \tilde{x}(t) = x(t) -\hat{x}(t). \end{aligned}$$

Applying $\mathscr {D}^\alpha$ in (36) and defining $\Gamma = B + L (\bar{D} - D)$, from (32), (35), (36) and replacing (25) for *u*(*t*), one arrives at

37 $$\begin{aligned}& \mathscr {D}^\alpha \tilde{x}(t)= \mathscr {D}^\alpha {x}(t) - \mathscr {D}^\alpha \hat{x}(t)=\bar{A} x(t) + \bar{B} u(t) - (A - LC) \hat{x}(t) - L \bar{C} x(t) - \Gamma u(t), \nonumber \\&=(\bar{A}- L \bar{C}) x(t) - (A - LC) \hat{x}(t) + (\bar{B} - \Gamma )u(t) =(\bar{A}- L \bar{C}) x(t) - (A - LC) (x(t) - \tilde{x}(t)) + (\bar{B} - \Gamma )u(t), \nonumber \\&=\big [(\bar{A}- L \bar{C}) - (A - LC) \big ] x(t) + (A - LC) \tilde{x}(t) + (\bar{B} - \Gamma )u(t) \\& = \big [(\bar{A}- L \bar{C}) - (A - LC) \big ] x(t) + (A - LC) \tilde{x}(t) + (\bar{B} - \Gamma ) [ - K_x (x(t) - \tilde{x}(t)) - K_{w_1} w_1(t) - K_{w_2} w_2(t)] \nonumber \\&= \big [(\bar{A}- L \bar{C}) - (A - LC) - (\bar{B} - \Gamma ) K_x \big ] x(t) + \big [A - LC + (\bar{B} - \Gamma ) K_x \big ] \tilde{x}(t) - (\bar{B} - \Gamma )(K_{w_1} w_1(t) + K_{w_2} w_2(t)). \end{aligned}$$

Substituting (25) and (33) into (21), it follows that

38 $$\begin{aligned} \mathscr {D}^{\alpha } w_1(t)&= (I_{q \times q} + K_r) (v(t) - \bar{C}x(t) - \bar{D}u(t)) - \varepsilon w_1(t) \nonumber \\&= (I_{q \times q} + K_r)v(t) - (I_{q \times q} + K_r)\bar{C}x(t) + (I_{q \times q} + K_r)\bar{D}K_x (x(t) - \tilde{x}(t)) + (I_{q \times q} + K_r)\bar{D}K_{w_1} w_1(t) \nonumber \\&\quad + (I_{q \times q} + K_r)\bar{D}K_{w_2} w_2(t) - \varepsilon w_1(t) \nonumber \\&= (I_{q \times q} + K_r)v(t) + (I_{q \times q} + K_r)(\bar{D}K_x - \bar{C}) x(t) - (I_{q \times q} + K_r)\bar{D}K_x \tilde{x}(t) + ((I_{q \times q} + K_r)\bar{D}K_{w_1} - \varepsilon I_{q \times q}) w_1(t) \nonumber \\&\quad + (I_{q \times q} + K_r)\bar{D}K_{w_2} w_2(t). \end{aligned}$$

In view of (22), (34), (38) and (39), the augmented pseudo-state equation becomes

39 $$\begin{aligned} \left[ {\begin{array}{*{20}c} {{\mathcal{D}}^{\alpha } x(t)} \\ {{\mathcal{D}}^{\alpha } w_{1} (t)} \\ {{\mathcal{D}}^{\alpha } w_{2} (t)} \\ {{\mathcal{D}}^{\alpha } \tilde{x}(t)} \\ \end{array} } \right] = & \left[ {\begin{array}{*{20}c} {(\bar{A} - \bar{B}K_{x} )} & { - \bar{B}K_{{w_{1} }} } & { - \bar{B}K_{{w_{2} }} } & {\bar{B}K_{x} } \\ {(I_{{q \times q}} + K_{r} )(\bar{D}K_{x} - \bar{C})} & {(I_{{q \times q}} + K_{r} )\bar{D}K_{{w_{1} }} - \varepsilon I_{{q \times q}} } & {(I_{{q \times q}} + K_{r} )\bar{D}K_{{w_{2} }} } & { - (I_{{q \times q}} + K_{r} )\bar{D}K_{x} } \\ {0_{{q \times n}} } & {I_{{q \times q}} } & { - \varepsilon I_{{q \times q}} } & {0_{{q \times n}} } \\ {(\bar{A} - L\bar{C}) - (A - LC) - (\bar{B} - \Gamma )K_{x} } & { - (\bar{B} - \Gamma )K_{{w_{1} }} } & { - (\bar{B} - \Gamma )K_{{w_{2} }} } & {p;A - LC + (\bar{B} - \Gamma )K_{x} } \\ \end{array} } \right]\left[ {\begin{array}{*{20}c} {x(t)} \\ {w_{1} (t)} \\ {w_{2} (t)} \\ {\tilde{x}(t)} \\ \end{array} } \right] \\ & + \left[ {\begin{array}{*{20}c} {0_{{n \times q}} } \\ {I_{{q \times q}} + K_{r} } \\ {0_{{q \times q}} } \\ {0_{{n \times q}} } \\ \end{array} } \right]v(t). \\ \end{aligned}$$

Finally, from (25) and (33), the output equation can be written as

40 $$\begin{aligned} y(t) = \bar{C}x(t) - \bar{D} K_x ( x(t) - \tilde{x}(t)) - \bar{D} K_{w_1} w_1(t) - \bar{D} K_{w_2} w_2(t)= (\bar{C} - \bar{D} K_x) x(t) + \bar{D} K_x \tilde{x}(t) - \bar{D} K_{w_1} w_1(t) - \bar{D} K_{w_2} w_2(t). \end{aligned}$$

In what follows, the synthesis of the controller gains $K_{xw}$ and *L* and the simulation of the closed-loop system under nominal conditions will be carried out by using the model (29), (30), whereas the simulations with parametric changes will be performed by using the model (40), (41).

## Results

The fractional order system was simulated from null initial conditions by using the ‘fractional integrator’ block of the FOMCON^61,62^ computational package, in the Simulink® environment of the MATLAB® software. All LMIs were solved with the YALMIP toolbox^63^ and SeDuMi solver^64^. The reference signal *v*(*t*) was set to a constant value during the inspiration phase^37^, p. 19-20, with a frequency of 16 cycles per minute, inspiration : expiration ratio of 1 : 2 and a flow setpoint of 480 ml per second. These settings were chosen to obtain a tidal volume of 600 ml and a minute volume of 9.6 litres per minute, which are adequate values for intermittent positive pressure ventilation^65^, p. 118. In the expiration phase, the input pressure was set to zero (i.e. ambient pressure), in order to obtain a passive outward flow.

We recall that the *A*, *B*, *C*, *D* matrices of the model (17), (18) are defined in (5), (6) with $N = 24$, resistances $R_1, R_2, \ldots R_{24}$ and capacitances $C_1, C_2, \ldots , C_{24}$ obtained as in (3), and experimental parameters $R_{UA}$, $C_{UA}$, $\alpha$ given in (4). Since $\alpha = 1.55$, the angle $\theta$ of the eigenvalue allocation region in Fig. 2 must be chosen in the range $(0, 0.225 \pi \,]$ radians, as discussed in Remark 2, which translates into a range of $(0, 40.5\,]$ degrees.

The gains $K_{xw}$ and *L* in $(A_{xwr} - B_{xwr}K_{xw})$ and $(A-LC)$ were obtained by setting $\varepsilon = 0.1$ in the leaky integrators (21), (22) and using Theorem 2. To this end, the following values were tested for the design parameters: $r \in \{100, 150, 200\}$, $\theta \in \{5, 10, 15\}$ degrees, and $K_r \in \{100, 300, 500\}$, yielding a total of 27 combinations. As a result, the settings that provided the smallest integral-time absolute error (ITAE)^66^ of the flow variable were $r = 200$, $\theta =5^{\circ }$, $K_r = 500$. Other values of $\varepsilon$ in the interval [0.01, 1] were also tested, but the ITAE results were similar, indicating that the choice of $\varepsilon$ was not critical for the design. The resulting gains $K_{xw}$ and *L* were

41 $$\begin{aligned} K_{xw}= \left[ \begin{matrix} -0.99901&-0.00031&-0.00012&-0.00004&0.00004&0.00011&0.00019&0.00027 \end{matrix} \right. \nonumber \\ \left. \begin{matrix} 0.00034&0.00040&0.00048&0.00060&0.00076&0.00099&0.00138&0.00192&0.00271 \end{matrix} \right. \nonumber \\ \left. \begin{matrix} 0.00380&0.00536&0.00779&0.01124&0.01621&0.02344&0.03094&-0.07292&-0.03209 \end{matrix} \right] , \end{aligned}$$

42 $$\begin{aligned} L= \left[ \begin{matrix} -22.0649&-7.3574&-5.1895&-3.8840&-3.0859&-2.5175&-2.0254&-1.7129&\end{matrix} \right. \nonumber \\ \left. \begin{matrix} -1.4046&-1.0638&-0.7863&-0.5355&-0.2966&-0.1130&0.0018&0.0898&0.0625&\end{matrix} \right. \nonumber \\ \left. \begin{matrix} -0.0085&-0.0781&-0.1399&-0.1727&-0.1766&-0.1854&-0.2080 \end{matrix} \right] ^{T}. \end{aligned}$$

**Fig. 5:**
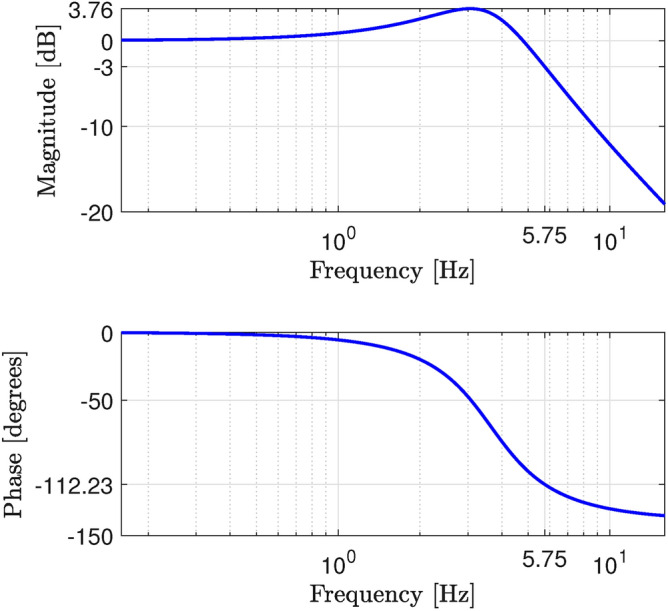
Frequency response of the closed-loop system.

As shown in Fig. 5, the passband of the resulting closed-loop system was approximately 6 Hz. Smaller ITAE values were obtained by reducing $\theta$ and/or increasing *r*, $K_r$, but these results were disregarded, as the resulting passband exceeded the range of frequencies in which the model was identified^25^.

**Fig. 6:**
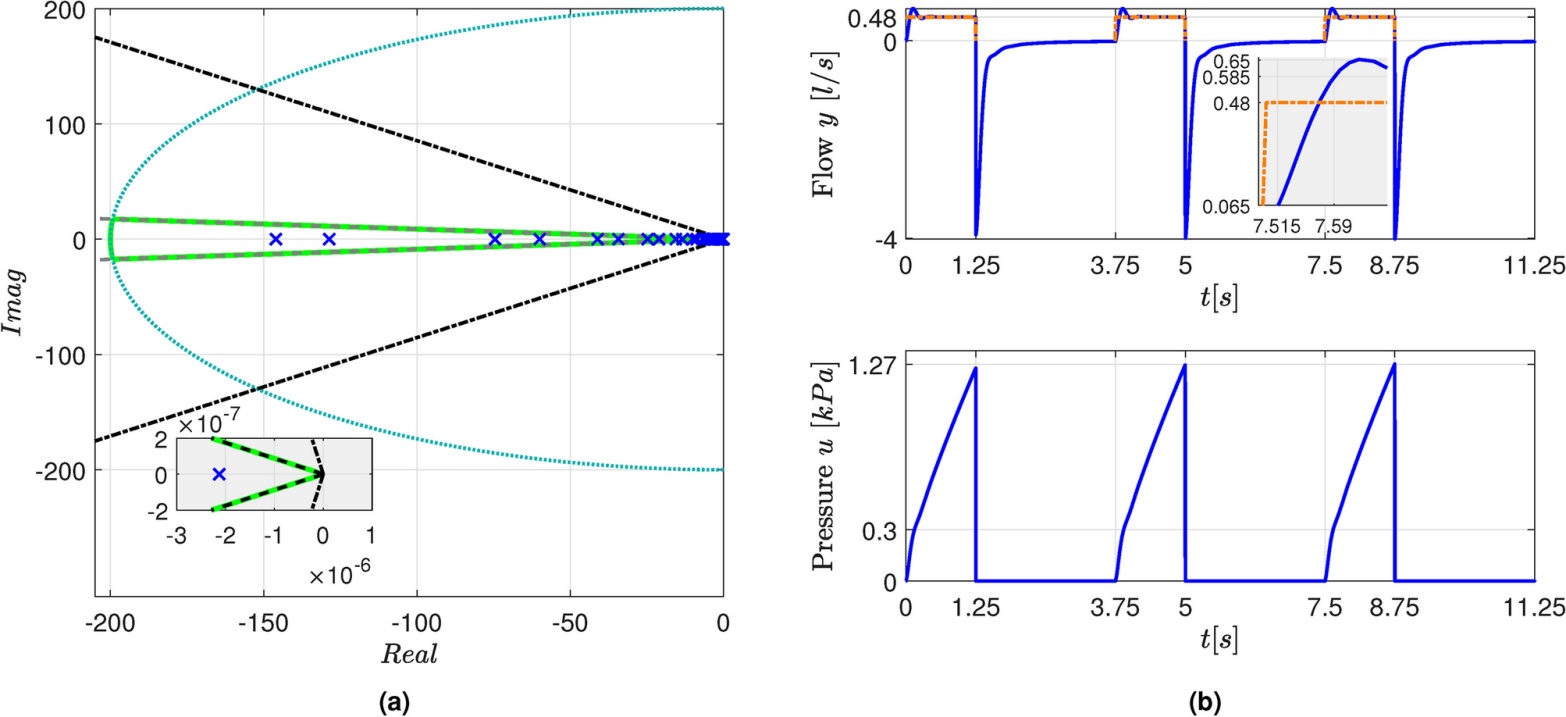
(**a**) Closed-loop eigenvalues (blue $\times$ markers) and boundary of the region $\mathfrak {D}$ region (green line). The boundaries associated to the $\theta$ and *r* parameters are indicated by the gray dashed lines and by the blue dotted circumference, respectively. The stability limits are indicated by black dash-dotted lines. The inset shows an enlarged view of the region close to the origin. (**b**) Simulation results. The *v*(*t*) reference for the flow is depicted by an orange dash-dotted line.

As shown in Fig. 6a, the closed-loop eigenvalues were all placed inside the region $\mathfrak {D}$ established in Theorem 2. Figure 6b presents the simulation results. As can be seen, a satisfactory tracking of the reference *v*(*t*) for the air flow was obtained.

**Fig. 7:**
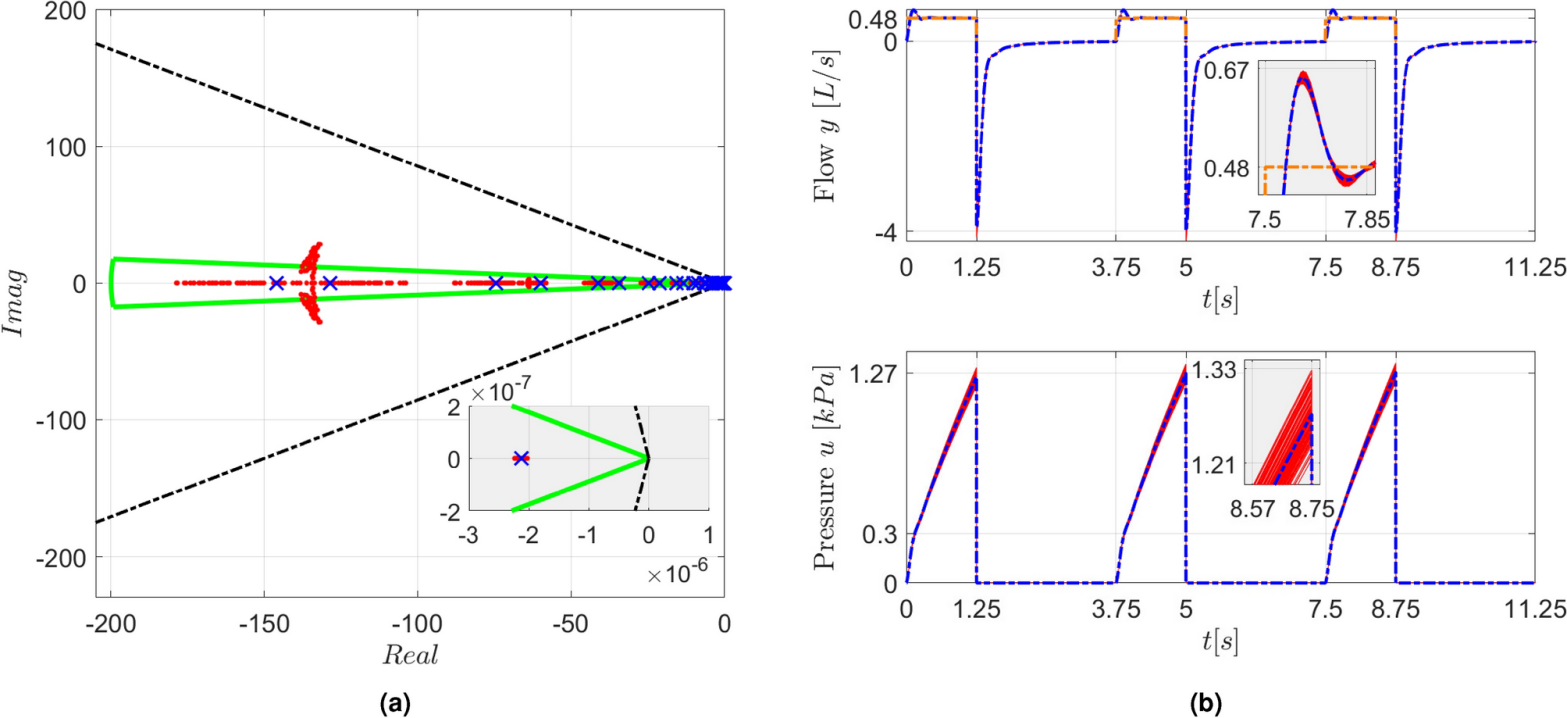
(**a**) Closed-loop eigenvalues for the nominal system (blue $\times$ markers) and for 100 perturbed systems with variations of up to $\pm 5\%$ in the $R_{UA}$ and $C_{UA}$ parameters (red $\bullet$ markers). The boundary of the $\mathfrak {D}$ region (green line) and the stability limits (black dash-dotted lines) are also shown. The inset shows an enlarged view of the region close to the origin. (**b**) Simulation results for the nominal (blue lines) and perturbed systems (red lines). The *v*(*t*) reference for the flow is depicted by an orange dash-dotted line. The insets present enlarged views of the dispersion around the nominal response.

**Fig. 8:**
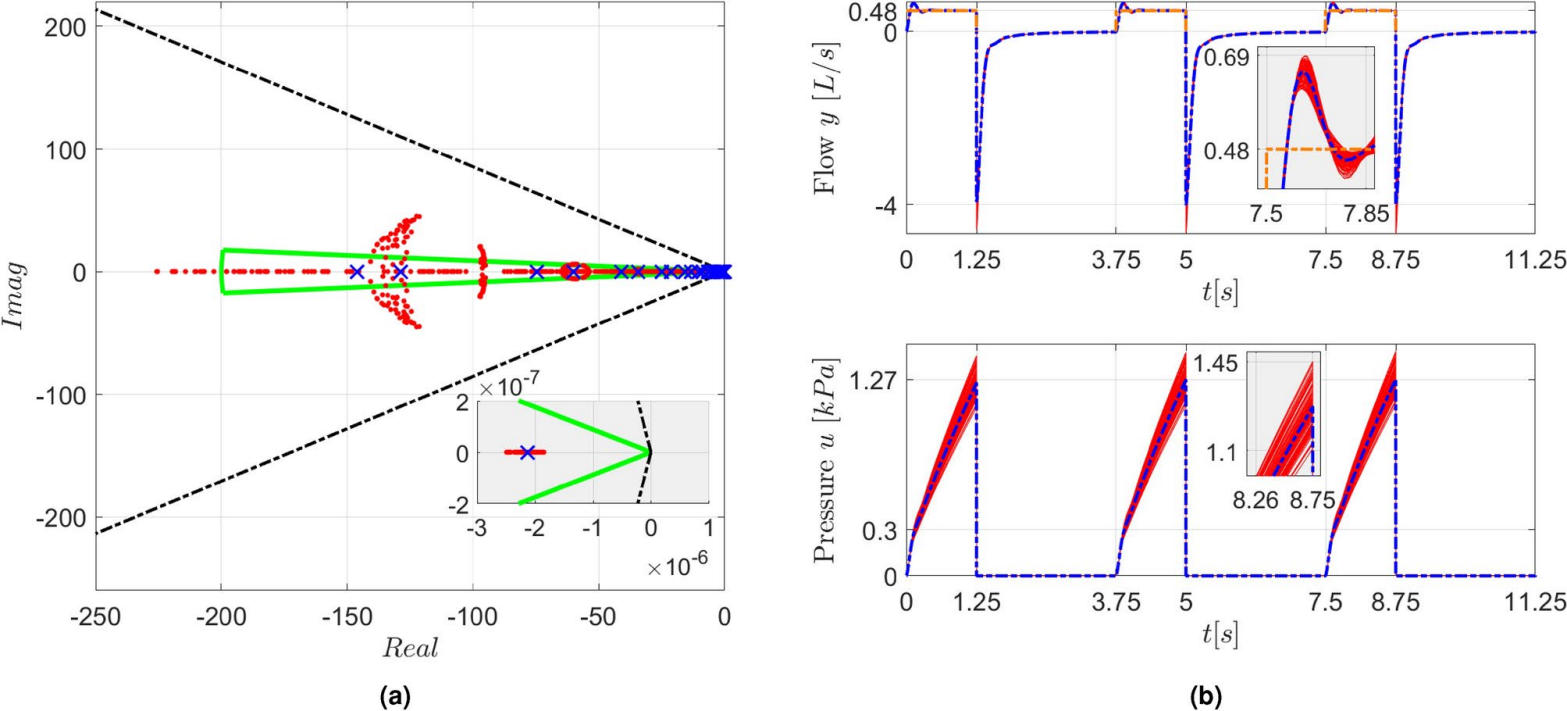
(**a**) Closed-loop eigenvalues for the nominal system (blue $\times$ markers) and for 100 perturbed systems with variations of up to $\pm 15\%$ in the $R_{UA}$ and $C_{UA}$ parameters (red $\bullet$ markers). The boundary of the $\mathfrak {D}$ region (green line) and the stability limits (black dash-dotted lines) are also shown. The inset shows an enlarged view of the region close to the origin. (**b**) Simulation results for the nominal (blue lines) and perturbed systems (red lines). The *v*(*t*) reference for the flow is depicted by an orange dash-dotted line. The insets present enlarged views of the dispersion around the nominal response.

The robustness of the closed-loop system was evaluated by randomly varying the $R_{UA}$ and $C_{UA}$ values, using a uniform probability distribution, in order to emulate uncertainties in the identification procedure or changes in the actual physiological parameters. The controller gains $K_{xw}$ and *L* were kept the same, whereas the simulation model was altered as indicated in (39) and (40) to reflect the changes in the $R_{UA}$ and $C_{UA}$ parameters. The closed-loop eigenvalues obtained for 100 parametric variations of up to $\pm 5\%$ and up to $\pm 15\%$ are shown in Figs. 7a and 8a, respectively. It is worth noting that some eigenvalues left the $\mathfrak {D}$ region, but all remained within the stability limits. The corresponding simulation results are presented in Figs. 7b and 8b, respectively. As can be seen, the parametric changes had some effect on the closed-loop response, but the rise time and overshoot did not deviate substantially from those obtained in the nominal case.

## Conclusions

The new formulations discussed in this work provide a design methodology for a controller whose feedback loop is implicit in a pseudo-state model, incorporating a Luenberger-type fractional-order linear observer and a double leaky integrator. Given the block-diagonal structure of the closed-loop system matrix in (29), the design of the gain matrices for the controller and observer can be carried out separately, with suitable placement of the eigenvalues in the stability domain defined for fractional systems. The regional allocation of eigenvalues over a specified region makes it possible to insert specifications for the system responses through LMI conditions. The methodology was applied to a well-accepted state-of-the-art electrical current recursive network model analogue of the human respiratory system and provided a pressure input capable of generating a desired airflow profile. In addition, by including uncertainties in the simulation model, the controller was found to be robust over a considerable range (up to $\pm 15 \%$) in the variation of the physiological parameters. Future steps for validating the proposed design methodology could include the use of a physical lung simulator with viscoelastic features^67^. Indeed, viscoelastic effects are known to be better described by fractional-order models as compared to integer-order representations^68–70^.

Although the current controller design does not explicitly take into account alveolar as well as alveolar dead space ventilation, the proposed fractional order model and the controller formulations may be augmented and appropriately adapted for such task. For example, such models could be used in conjunction with real-time advances in magnetic resonance imaging (MRI) using hyperpolarized Xenon (Xe)-129 or Helium-3^71,72^, which can provide improved in situ real-time diagnosis of a variety of lung diseases such as emphysema and chronic obstructive pulmonary disease (COPD) thus providing new opportunities for dynamic ventilation.

A further possible merit of adopting a fractional order modelling and control methodology of the lung impedance is that it may also be used to assess levels of injury (barotrauma, volutrauma, atelectrauma, and biotrauma) or assess the progression of regional lung strain associated with mechanical ventilation as opposed to spontaneous breathing^73^. This is particularly important to patients with acute respiratory distress syndrome (ARDS) where lung tissue undergoes additional regional stress and modification due to inflammation under prolonged mechanical ventilation. With minor modifications to the generic controller structure, the methodology developed can be augmented to account explicitly for additional input parameters such as ${\textrm{CO}}_2$ partial pressure levels or partial oxygen saturation levels so that a larger application domain can be considered (e.g. application to extracorporeal membrane oxygenation (ECMO) machines accounting also for decarboxylation. In addition, oxygen release curves (partial pressure of oxygen as a function of saturation of haemoglobin) as affected by the Bohr effect, changed pH or diphosphoglycerate (DPG) levels when monitoring maternal and foetal gas exchange can be also considered in the model. The associated Hill plots accounting for cooperative oxygen binding to haemoglobin are well known for their fractional order characteristics and can be explicitly translated to controller parameter specifications. Finally, it is worth also noting that in order to address issues of progression of regional lung strain associated with mechanical ventilation, it should be possible to develop a more adaptive control strategy by assuming an internal model control loop with external adaptive control. Such schemes have already been extended to fractional order systems^74^, but have yet to be explored in the context of lung ventilation on the basis of the new formulations we have presented in this paper.

The proposed control methodology is generic and applicable to a wide range of biomedical control problems as well as for the control of complex physicochemical systems. Future work could investigate the use of the proposed methodology in applications such as the control of current flow across large-scale electrical networks^75^, the deformation of viscoelastic materials^76^, and the charging process of energy storage devices^77^.

## Data Availability

All data generated or analysed during this study are included in this published article.
